# Mapping headphone brand positioning from e-commerce reviews with large language models

**DOI:** 10.1371/journal.pone.0361055

**Published:** 2026-11-11

**Authors:** Jian Li, Hengshan Cao

**Affiliations:** School of Business, Wuxi Taihu University, Wuxi, Jiangsu, China; Philadelphia University, JORDAN

## Abstract

Online consumer reviews encode rich signals about brands, yet converting this unstructured text into actionable positioning insight remains challenging. This study proposes an LLM-driven framework that extracts structured brand perception variables from e-commerce reviews and demonstrates it in the headphone category. Using an analytic file of 5,895 review-level observations covering Sony, Bose, JBL, and Beats, we employ a structured GPT-5.4 prompt to extract multidimensional brand perception indicators from each review, capturing associations, judgments, emotional attachment, advocacy, and attribute focus. A manual validation exercise based on 400 stratified reviews showed meaningful agreement between the LLM-coded outputs and an adjudicated human benchmark. The results reveal clear brand separation. Bose leads on perceived quality (4.20/5), recommendation rate (78.3%), and strong emotional connection (39.9%), whereas Sony achieves the highest value perception (3.97/5). Beats and JBL occupy weaker quality positions, though Beats retains a comparatively stronger emotional profile. Patterns in attribute emphasis also help distinguish the brands: Bose reviews are distributed across sound quality, active noise cancellation, and comfort; Sony reviews emphasize sound quality, comfort, and value; JBL reviews are heavily concentrated around sound quality; and Beats reviews emphasize sound quality, value, and design. Negative reviews provide additional diagnostic insight, revealing durability and fit issues for JBL and perceived value concerns for Beats. The study contributes primarily to LLM-assisted marketing analytics and applied brand diagnosis by showing how constrained and human-validated LLM coding can transform review text into a scalable, multidimensional brand monitoring instrument. Brand equity and positioning theory are used as conceptual foundations for organizing the extracted perception variables rather than as targets of theoretical extension.

## Introduction

Brand strategy rests on understanding how consumers interpret and compare competing offerings. In digital commerce, much of this interpretive work now unfolds publicly through product reviews, ratings, and recommendation behavior. Beyond indicating satisfaction, reviews reveal which dimensions of the brand experience consumers prioritize, how they weigh competing attributes, and where rival brands are positioned in the consumer mind [1,2]. Capitalizing on this potential, however, poses substantial methodological challenges. Survey-based brand research is valuable yet expensive, episodic, and often detached from the language consumers naturally use [3,4]. Simple sentiment analysis scales well but compresses rich evaluations into coarse polarity scores, offering limited insight into positioning or association structure [5]. Text-mining approaches have shown that online discourse can reveal market structure and brand meaning [6,7], yet many require extensive preprocessing, labeled data, or narrow task definitions. This challenge is amplified as digital channels render brand meaning more observable yet less stable. Reviews accumulate continuously, blending functional and symbolic judgments and frequently drawing direct comparisons across competing brands. In digitally mediated categories such as headphones, where consumers simultaneously evaluate sound, comfort, style, price, and ecosystem compatibility, review text constitutes a high-frequency record of competitive positioning that conventional research methods seldom capture [4,8]. The emergence of large language models (LLMs) offers a promising avenue for addressing these limitations. Their capacity to follow structured prompts, synthesize nuanced text, and produce normalized outputs without dedicated fine-tuning makes them a promising instrument for converting review narratives into analyzable brand perception indicators [9,10]. Recent marketing research has begun to validate LLMs for perceptual analysis, hybrid marketing research workflows, and customer-need extraction [11–14]. Recent marketing research has also begun to describe a broader shift from isolated automation tasks toward richer analytical applications grounded in customer language and marketplace data [15–17]. However, prior research has mainly examined simulated respondents or narrowly defined analytical tasks, providing limited evidence on how LLMs can support comprehensive and comparative brand diagnostics based on naturally occurring consumer reviews. This study addresses that gap through a comparative investigation in the headphone category. Using outputs from a structured LLM prompt applied to reviews of Sony, Bose, JBL, and Beats, we compare the four brands across quality, value, emotional connection, recommendation, brand association, and primary attribute focus. Rather than positioning LLMs as replacements for conventional brand research methods, this study conceptualizes them as complementary analytical instruments that can extend existing approaches by translating large volumes of consumer review language into scalable insights into competitive brand positioning.

To guide this investigation, three research questions are posed:

- **RQ1:** Can a structured LLM prompt transform unstructured headphone reviews into usable, analytically comparable brand perception variables?
- **RQ2:** Do the extracted variables reveal meaningful differences in competitive brand positioning across Sony, Bose, JBL, and Beats?
- **RQ3:** What strategic implications emerge from the integration of perceptual maps, attribute-focus patterns, and negative review diagnostics?

In addressing these questions, this study contributes to the literature by linking CBBE theory, LLM-enabled marketing analytics, and competitive positioning analysis. First, the study uses CBBE and positioning theory as conceptual foundations for organizing review-based perception variables rather than claiming to extend brand equity theory itself. These conceptual variables include perceived quality, value perception, emotional connection, recommendation intent, brand association, and attribute focus [1,3].This framing allows naturally occurring review narratives to be converted into structured indicators that support scalable and comparative brand-positioning analysis. Second, it advances emerging work on LLM-based marketing analytics by embedding structured extraction within a theory-guided comparative framework, rather than treating LLM outputs as standalone summaries or unconstrained textual interpretations [11,18]. Third, it offers a reproducible workflow through which platform reviews can be used as a continuing source of competitive brand-positioning diagnostics. Fig 1 provides an overview of the proposed framework, illustrating how review text is transformed into structured brand perception variables and competitive positioning diagnostics.

**Fig 1:**
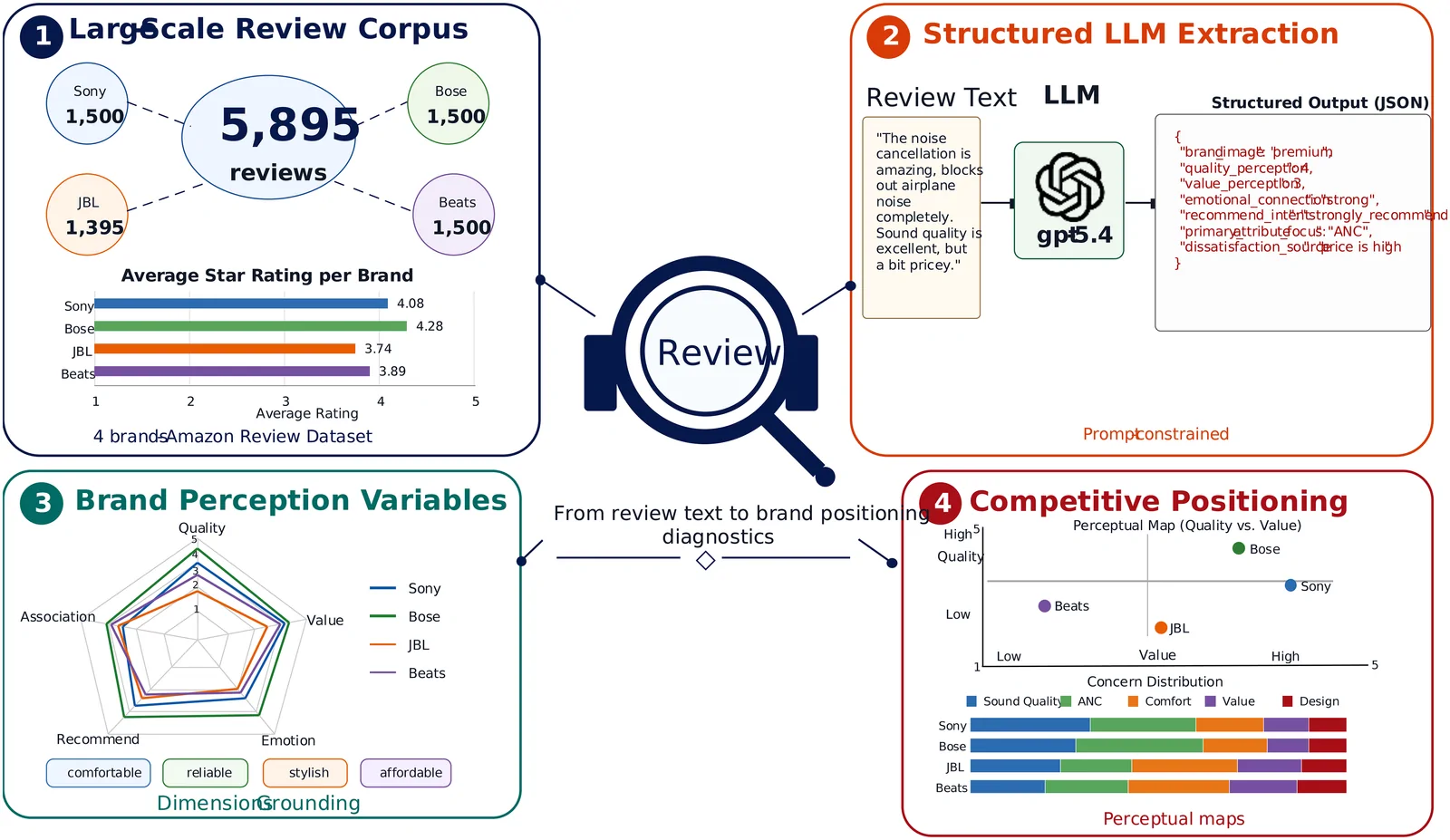
Overview of the proposed LLM-driven brand positioning framework.

## Literature review

### Brand perception, brand equity, and positioning

Brand perception encompasses the constellation of associations, evaluative judgments, and relational signals that consumers form around a brand. Within the CBBE tradition, strong brands derive their advantage not merely from awareness but from favorable, distinctive associations, positive performance and imagery judgments, and deeper affective bonds between consumers and the brand [1,3]. Aaker’s brand equity framework reinforces this view by identifying perceived quality, brand associations, and loyalty as core intangible assets that require continuous measurement and strategic management [2]. A central implication of these frameworks is that brand analysis must be inherently multidimensional. An exclusive focus on aggregate satisfaction can mask important differences across perception dimensions, such as high perceived quality coupled with weak value perceptions, strong emotional appeal coupled with inconsistent usage experiences, or recommendations that vary across use contexts. Competitive positioning, accordingly, depends not on any single attitude score but on how multiple perception dimensions cohere and diverge across rival brands within a shared market space.

### Text analytics and review mining in marketing

Marketing scholars have long recognized text produced by users as a valuable source of brand insight, given its capacity to capture spontaneous language, granular consumption experiences, and naturally occurring comparisons across products [4]. Early automated approaches relied on sentiment classification and coding using dictionaries, whereas later work introduced topic models and more flexible machine learning methods that could infer market structure and brand meaning from textual data [5–7]. Timoshenko and Hauser [19] further demonstrated that content created by users can serve as a valuable input for identifying latent customer needs. Despite these advances, conventional text analytics pipelines involve persistent tensions between interpretability, annotation burden, and domain adaptation. Transformer architectures such as BERT improved classification performance considerably [20], yet applied brand research settings still frequently demand additional labeling, custom modeling, or substantial technical expertise. This creates a widening gap between methodological possibility and managerial usability. In applied brand research, this gap is particularly consequential because managers require outputs that are transparent, repeatable, and aligned with recognizable constructs such as quality, value, comfort, and recommendation, rather than relying solely on opaque latent dimensions. A further limitation of earlier review mining work is that many systems are optimized for either predictive accuracy or broad thematic discovery, but not for competitive brand diagnosis. Positioning analysis demands outputs that are directly comparable across brands, interpretable by nontechnical stakeholders, and stable enough to support repeated tracking. These requirements also help explain why many firms continue to rely on manual coding, sentiment dashboards, or external research providers, even as large volumes of review text have become increasingly accessible.

### Large language models in marketing research

Generative AI has broadened the scope of feasible marketing research tasks by enabling structured reasoning over unstructured language [10,21,22]. In applied research settings, LLMs have been used to simulate respondents, assist qualitative interpretation, and automate perceptual analysis [11–13], while recent evidence suggests that they can approximate performance comparable to that of experts in extracting customer needs from text under certain conditions [14]. At the same time, review analysis studies caution that task design and benchmarking remain critical, and that LLMs should be evaluated against simpler alternatives rather than adopted uncritically [23,24]. This emerging literature suggests that LLMs are particularly valuable in brand management when they are used not for unconstrained generation, but for structured extraction within a predefined analytical schema. Such a design constrains output variability, supports comparability across brands, and aligns the method with managerial decision needs. Adjacent work on consumer segmentation and review-based construct modeling reinforces this view, showing that LLMs can translate messy, naturalistic language into theoretically meaningful categories [25,26], while comparative studies in electronic commerce demonstrate that prompt design, label discipline, and evaluation strategy strongly affect output quality [27]. Collectively, these findings suggest that the value of LLMs in marketing lies less in replacing theory than in making theory-guided coding more scalable.

Recent studies have emphasized the importance of how reviews are sampled, how language models are compared, and how customer reviews can support comparisons between products and services. One study shows that selecting reviews through stratified sampling can improve the balance and efficiency of summaries, suggesting that the sampling procedure affects which aspects of consumer opinion are retained [28]. Another compares GPT and LLaMA for evaluating product reviews and demonstrates why model choice and systematic benchmarking matter [27]. A third examines how generative AI can use customer reviews [29]to compare products and services. The present study differs in its focus on a schema grounded in theory that converts review text into variables describing how consumers perceive brands, compares competing brands, and identifies recurring sources of dissatisfaction. Its contribution lies in combining structured coding, human validation, and interpretation of competitive brand positions, rather than focusing only on summarizing reviews or comparing language models.

### Research gap

Despite rapid progress, three notable gaps remain in the literature. First, much of the published work prioritizes synthetic respondents or methodological validation over direct competitor analysis drawn from real review corpora [12,13]. Second, existing applications tend to address one analytical layer at a time, whether sentiment, customer needs, or perceptual similarity, rather than integrating multiple brand perception dimensions within a unified framework [11,14]. Third, limited evidence exists on how LLM-coded review data can be translated into actionable, brand-level positioning implications. The present study addresses these gaps by applying a structured LLM coding workflow to real headphone reviews and linking the extracted outputs to brand comparison tables, perceptual maps, attribute-focus analysis, and strategic interpretation. To position the contribution of this study more clearly, Table 1 contrasts the main streams of prior research with the remaining limitations addressed here.

**Table 1:** Representative streams of prior research and the gap addressed in this study.

Study stream	Typical data source	Main contribution	Remaining limitation for brand strategy
Text mining for marketing insight [4,6,7]	Online text, forums, reviews	Reveals themes, market structure, and brand meaning	Often requires specialized modeling and does not directly yield manager-accessible perception constructs
Transformer and NLP classification approaches [5,20]	Labeled text corpora	Improves text classification and sentiment inference	High setup burden for multidimensional competitive brand diagnosis
LLM perceptual and market research applications [11–13]	Simulated queries, hybrid research workflows	Validates LLMs for perceptual analysis and insight support	Limited direct application to multibrand diagnosis from real marketplace reviews
LLM review and insight extraction studies [14,25,26]	Real reviews and consumer text	Shows that LLMs can derive structured constructs from text	Often focuses on one construct family or noncomparative brand settings
**This study**	**Real headphone reviews**	**Uses one structured schema to compare four competing brands**	**Demonstrates an integrated workflow for positioning, attribute-focus analysis, and dissatisfaction diagnosis**

### Theoretical framing

The study is grounded in the view that brand equity is embedded in consumers’ knowledge structures and expressed through the associations, judgments, and relationship signals attached to a brand. In Keller’s CBBE framework, brand strength derives from what consumers know about a brand, how they evaluate it, and the forms of response and attachment it elicits [1,3]. Translating this logic to review data, the analysis treats brand image labels and attribute focus as indicators of salient associations, quality and value scores as indicators of evaluative judgment, emotional connection as an indicator of relational intensity, and recommendation intent as a behavioral advocacy signal. These measures are not intended to replicate validated survey scales; rather, they serve as review-based indicators that correspond to the theoretical domains emphasized in CBBE. A second theoretical premise is that positioning is relational. A brand’s meaning and strength become strategically relevant only when interpreted against alternatives competing for similar use occasions, symbolic meanings, and consumer budgets. For this reason, the analysis focuses on cross-brand comparison rather than isolated review summarization. The central question is not simply how consumers describe Bose, Sony, JBL, or Beats individually, but how patterns of evaluation and association distinguish these brands within a shared competitive space. This comparative orientation follows marketing research that treats perceptual space and market structure as inherently relative phenomena [6,11]. The third premise is that platform reviews function as digital traces of marketplace sensemaking. Reviews record what consumers notice, praise, question, and compare as they make sense of products in use. They are therefore more than postpurchase satisfaction statements; they provide distributed evidence of how brand meaning is negotiated in the market. LLM-based coding is useful in this setting because it can transform large volumes of narrative evidence into structured, comparable variables at a scale that manual coding rarely supports [10,21,30]. Viewed this way, the study positions generative AI as part of a broader shift from episodic brand measurement toward AI-assisted market sensing grounded in naturally occurring consumer language. Table 2 summarizes how the study variables map onto these theoretical domains and how each set of variables is used analytically.

**Table 2:** Mapping the study variables to the underlying theoretical framework.

Theoretical lens	Study variables	Interpretive role in the analysis
Brand meaning and associations	brand image; primary attribute focus	Captures what the brand is perceived as and which attribute family dominates consumer attention
Brand judgments	quality perception; value perception	Captures evaluative assessment of performance and price justification
Brand relationships	emotional connection; recommendation intent	Captures attachment intensity and advocacy likelihood
Competitive positioning	perceptual maps; comparative brand analysis	Converts brand-level coded outputs into a relative market space interpretation
Digital market sensing	negative review diagnostics; attribute-focus distribution	Identifies recurring friction and emergent vulnerabilities from naturally occurring text

This approach provides a structured observational method for identifying strategically meaningful patterns in review language, supporting comparative diagnosis, repeated tracking, and closer alignment between brand analysis and the language consumers use in the marketplace.

## Materials and methods

### Data source and analytic sample

The study draws on the headphone segment of the Amazon review ecosystem. The underlying data source is the Amazon Review Dataset compiled by Ni, Li, and McAuley [31]. For the present analysis, we employed a filtered subset of headphone reviews covering four focal brands: Sony, Bose, JBL, and Beats. These brands were selected on the basis that they are major competitors in consumer audio and represent meaningfully distinct market positions in terms of premium reputation, feature emphasis, lifestyle symbolism, and value appeal. To improve comparability across brands, reviews were restricted to headphone products while non-headphone accessories or unrelated consumer electronics items were excluded where identifiable from product title and categories. When available, product identifiers and titles were utilized to verify that the retained reviews corresponded strictly to the target hardware rather than unrelated accessories or non-comparable product lines. The analytic file used in this study comprises 5,895 observations processed via the LLM extraction pipeline. The sampling process capped the target at 1,500 reviews per brand where available; the final counts are 1,500 reviews each for Sony, Bose, and Beats, and 1,395 for JBL. For records with available review-date metadata, the retained reviews covered the period from January 13, 2003 to September 28, 2018. Table 3 summarizes the analytic sample.

**Table 3:** Analytic sample overview by brand.

Brand	Reviews	Avg. rating	Negative share (%)	Avg. review length
Sony	1,500	4.08	25.5	154.4
Bose	1,500	4.28	19.5	153.7
JBL	1,395	3.74	36.6	155.7
Beats	1,500	3.89	31.3	129.6
**Total / Avg.**	**5,895**	**4.00**	**28.1**	**148.2**

Duplicate review records were checked and removed where identified before LLM processing. Product-level heterogeneity was also considered during filtering to reduce the risk that clearly distinct product types or substantially different product versions would distort cross-brand comparability. Negative share is defined as the proportion of reviews receiving a star rating of three or below. Average review length is reported in characters. These descriptive statistics indicate nontrivial variation across brands. Bose exhibits the strongest rating profile, JBL the weakest, while Beats and Sony occupy intermediate positions. The sampling procedure extracted reviews brand by brand rather than from the pooled corpus, with a target of 1,500 observations per brand, ensuring that no single brand dominates the analytic set by virtue of higher review volume. The category focus on headphones is analytically advantageous because the category simultaneously engages functional, experiential, and symbolic evaluation dimensions, making it well suited for a multidimensional brand perception analysis. Because brand positioning in consumer electronics may be influenced by product generation, pricing tier, and market timing, these results represent a cross-sectional snapshot of online reviews rather than a static assessment of brand equity. Although our filtering protocol mitigated blatant category mismatches, residual variations across product models, generations, and review windows may still influence the perceived positioning, which should be taken into account when interpreting the brand-level comparisons.

The quota-based sampling design was intended to support balanced cross-brand comparison rather than to reproduce the population-level distribution of consumer reviews. Equalizing the approximate number of reviews across brands does not guarantee an equivalent distribution of positive, neutral, and negative opinions. In the analytic sample, the observed negative-review share varied across brands, ranging from 19.5% for Bose to 36.6% for JBL. Accordingly, the brand-level results should be interpreted as comparisons conditional on the analytic sample, rather than as estimates of the prevalence of consumer opinions in the broader market.

### LLM-based extraction, validation, and analysis

Brand perception variables were derived from a structured prompt administered via GPT-5.4, with each review submitted to the model alongside the corresponding brand name, star rating, and product title. For reproducibility, each review was processed independently using the same fixed prompt template and constrained JSON output schema. The extraction used the same input fields for all records, including brand name, review text, star rating, and product title, and no brand-specific prompt adaptation was applied. The model identifier, system-instruction setting, decoding parameters, and output validation procedure are reported in S1 Appendix. Outputs that were unparseable or deviated from the predefined JSON schema were corrected using the same uniform procedure across all brands. To ensure comparability across brands, the output space was constrained through predefined vocabularies and restricted category taxonomies. The extracted dimensions included brand image, quality perception, value perception, emotional connection, recommendation intent, primary attribute focus, and dissatisfaction source. Table 4 summarizes the extracted dimensions.

**Table 4:** Extracted brand perception dimensions.

Dimension	Operationalization	Strategic meaning
Brand image	One controlled descriptor from the prompt vocabulary	Salient symbolic or evaluative association attached to the brand
Quality perception	Integer score from 1 to 5	Perceived product performance and overall quality judgment
Value perception	Integer score from 1 to 5	Perceived price justification assessment
Emotional connection	Weak / moderate / strong	Strength of affective attachment signaled in the review
Recommendation intent	Strongly recommend / recommend / neutral / not recommend	Behavioral advocacy signal
Primary attribute focus	One label from the attribute-focus taxonomy	Main product attribute emphasized by the reviewer
Dissatisfaction source	Short free-text issue phrase or *none*	Specific source of friction in negative or mixed experiences

To evaluate the reliability of the LLM-coded variables, we conducted a manual annotation validation study. A stratified random sample of 400 reviews was drawn from the analytic corpus, including 100 Sony reviews, 100 Bose reviews, 100 JBL reviews, and 100 Beats reviews. The validation sample was designed to include positive, mixed, and negative reviews so that the coding task covered a range of evaluative tones. Two independent coders annotated each review using the same conceptual schema as the LLM extraction task, including quality perception, value perception, emotional connection, recommendation intent, primary attribute focus, and dissatisfaction source. Coders were blind to the LLM outputs and to the aggregate brand-level results. Disagreements between coders were resolved through discussion to create an adjudicated human benchmark. Inter-coder reliability was assessed before adjudication. For the ordinal quality and value scores, we report quadratic weighted Cohen’s kappa, Spearman’s rho, and mean absolute error. For emotional connection and recommendation intent, human-human reliability was summarized using weighted Cohen’s kappa, while LLM-human classification agreement was summarized using Cohen’s kappa, accuracy, and macro-F1. For primary attribute focus, we report Cohen’s kappa, accuracy, and macro-F1. The LLM outputs were then compared with the adjudicated human benchmark using the same validation logic. After validation, the extracted variables were aggregated at the brand level to compare quality perception, value perception, recommendation intent, and emotional connection across Sony, Bose, JBL, and Beats. These aggregate indicators were then used to construct perceptual maps and were supplemented with brand-image labels, attribute-focus distributions, and recurring dissatisfaction themes from negative reviews. The aggregation strategy was kept deliberately simple to prioritize interpretability, transparency, and replicability.To evaluate whether structured extraction via large language models provides incremental analytical value relative to simpler techniques for review analysis, we conducted a baseline comparison. This approach aligns with recent methodological recommendations that coding derived from large language models should be benchmarked against simpler alternatives rather than adopted uncritically. Two deterministic baselines were specified. The first treated the observed star rating as a direct prediction of quality perception on a scale from one to five, deriving the classification for recommendation intent through a predefined mapping rule: a rating of 5 indicated a strong recommendation, 4 a recommendation, 3 a neutral stance, and 1 or 2 a negative recommendation. The second approach scored each review utilizing the VADER sentiment analyzer [32], which relies on lexicons and rules optimized for text generated by users. The resulting compound polarity score was mapped to the four recommendation categories through fixed thresholds, where scores of at least 0.60 signified a strong recommendation, scores from 0.05 to 0.60 a recommendation, scores from −0.05 to 0.05, a neutral stance −0.05 a negative recommendation. Both rules are deterministic and were applied uniformly across all brands. Both rules are deterministic and were applied uniformly across brands. Baseline outputs were compared with the variables coded by the language model across the full analytic corpus of 5,895 reviews. For ordinal scores, we calculated quadratic weighted Cohen’s kappa, Spearman’s rank correlation, and mean absolute error; for categorical labels, we employed Cohen’s kappa, classification accuracy, and macro-F1 scores. Because the agreement between model outputs and the adjudicated human benchmark was established independently in the validation study, high convergence between a baseline and the model outputs on a given dimension indicates that the dimension is recoverable without structured extraction. Conversely, dimensions for which the baselines produce no output highlight the structural contribution of the framework. Although topic modeling and clustering through embeddings were additionally considered, their unsupervised outputs do not correspond to the predefined taxonomy of brand perception. Consequently, they cannot be evaluated on the same prediction task and are instead examined as qualitative contrasts. The objective of this analysis is not to establish that structured extraction supersedes conventional techniques, but rather to delineate which dimensions of brand perception are already recoverable from simpler evaluative signals and which necessitate structured extraction.

## Results

### Validation of LLM-coded variables

Before aggregating the LLM-coded variables to the brand level, we evaluated their agreement with human annotations. Table 5 reports the validation results. Inter-coder reliability was moderate to substantial across the main coding dimensions, indicating that the coding schema could be applied consistently by human annotators. The LLM-human comparison showed the strongest agreement for recommendation intent, followed by quality perception and primary attribute focus, while agreement was weaker but still acceptable for value perception and emotional connection. These results support the use of the LLM-coded variables for aggregate brand-level descriptive analysis, while indicating that fine-grained interpretations of emotional connection and value perception should be treated with caution.

**Table 5:** Human validation of LLM-coded variables.

Variable	Human-human reliability	LLM-human agreement	Interpretation
Quality perception	weighted κ = 0.74; ρ = 0.81	weighted κ = 0.68; ρ = 0.76; MAE = 0.52	Substantial agreement; LLM scores track human ratings closely with approximately half-point average deviation
Value perception	weighted κ = 0.66; ρ = 0.74	weighted κ = 0.59; ρ = 0.68; MAE = 0.63	Acceptable agreement; value judgments are more subjective than quality judgments
Emotional connection	weighted κ = 0.62	κ = 0.57; accuracy = 0.73; macro-F1 = 0.69	Moderate agreement; the moderate category absorbs much of the disagreement in affective intensity
Recommendation intent	weighted κ = 0.78	κ = 0.71; accuracy = 0.79; macro-F1 = 0.74	Substantial agreement; recommendation intent aligns closely with explicit review cues
Primary attribute focus	κ = 0.64	κ = 0.61; accuracy = 0.71; macro-F1 = 0.63	Substantial agreement given the nine-class taxonomy; most disagreement occurs between adjacent attribute categories

### Baseline comparison

Table 6 reports the comparison between the two deterministic baselines and the structured LLM extraction. Because the agreement between the LLM outputs and the adjudicated human benchmark is established in the preceding subsection, the baseline comparison addresses two complementary questions: which extracted dimensions are already recoverable from signals present in the raw data, and which dimensions the baselines cannot produce at all.

**Table 6:** Deterministic baseline comparison with the structured LLM extraction.

Baseline	Result	Interpretation
Star rating as quality prediction	Agreement with LLM quality perception: weighted κ = 0.935; ρ = 0.914; MAE = 0.201. Identical brand ranking (Bose > Sony > Beats > JBL).	Overall quality is largely recoverable from the star rating alone; the convergence is expected by design because the rating is supplied to the model as contextual input. No added value is claimed for this dimension.
Star rating mapped to recommendation intent	Agreement with LLM recommendation intent: κ = 0.771; accuracy = 0.851; macro-F1 = 0.792.	Coarse advocacy classification is attainable from the rating, but the rule cannot indicate why consumers endorse or reject a brand.
VADER sentiment polarity	Correlation with LLM quality: ρ = 0.367; with LLM value: ρ = 0.339. Mapped recommendation: κ = 0.255; accuracy = 0.551; macro-F1 = 0.379.	A unidimensional polarity score neither separates quality from value judgments nor reproduces the advocacy classification.
Structural coverage of both baselines	No output for value perception as a distinct variable, brand image, emotional connection, primary attribute focus, or dissatisfaction source.	These dimensions, including the value ranking that reverses the quality ranking (Sony > Bose; JBL > Beats), are available only through structured extraction.

For overall quality, the star rating is largely sufficient. LLM quality scores track star ratings closely (weighted κ = 0.935; ρ = 0.914; MAE = 0.201), and both measures induce the identical brand ranking (Bose > Sony > Beats > JBL; Tables 3 and 7). This convergence is expected by design, since the star rating is supplied to the model as contextual input, and we accordingly make no claim that the framework adds value in measuring overall quality. The fixed star-to-recommendation mapping similarly reproduces most of the extracted recommendation intent (κ = 0.771; accuracy = 0.851; macro-F1 = 0.792), indicating that coarse advocacy classification is also attainable without structured extraction.

**Table 7:** Brand perception comparison across key dimensions.

Brand	Quality (1–5)	Value (1–5)	Strong emotion (%)	Recommend rate (%)	Salient association	Leading attribute focus(es)
Sony	3.87	3.97	28.3	73.3	reliable	sound quality
Bose	4.20	3.89	39.9	78.3	comfortable	sound quality / ANC
JBL	3.63	3.59	28.8	60.6	affordable	sound quality
Beats	3.72	3.41	30.9	65.2	stylish	sound quality

The added value of the framework emerges on the dimensions that a single evaluative score cannot represent. The VADER compound polarity shows weak and nearly identical correlations with the extracted quality (ρ = 0.367) and value (ρ = 0.339) scores, and its threshold-based mapping reproduces the recommendation classification far less closely (κ = 0.255; accuracy = 0.551; macro-F1 = 0.379), indicating that a unidimensional polarity signal neither separates performance judgments from price-justification judgments nor recovers the advocacy structure. More fundamentally, neither baseline can produce value perception as a variable distinct from quality, nor brand image, emotional connection, primary attribute focus, or dissatisfaction source; for these fields the limitation is structural rather than a matter of accuracy. The consequence is visible at the brand level: whereas star ratings and LLM quality order the four brands identically, the value dimension reverses both pairs (Sony > Bose and JBL > Beats; Table 7), a positioning-relevant distinction that no star-based or polarity-based measure can generate. Attribute-focus distributions (Table 9) and dissatisfaction themes (Table 10) likewise depend on the structured extraction. Taken together, the comparison indicates that the contribution of the framework lies not in re-estimating overall evaluative tone, which star ratings already capture, but in decomposing review language into the multidimensional positioning variables on which the competitive analysis below is based.

Comparisons were computed on the full analytic corpus (n = 5,895). Agreement between the LLM outputs and the adjudicated human benchmark is reported in Table 5.

### Brand-level perception and positioning

After the validation step, Table 7 reports the core brand perception metrics. Bose leads the category on perceived quality, recommendation rate, and strong emotional connection, while Sony records the highest value perception and the second highest recommendation rate, suggesting a strong position grounded in functional performance and value. Beats and JBL trail on average quality, with JBL exhibiting the weakest overall rating and the highest share of negative reviews. Beats, however, outperforms JBL on both quality and recommendation while generating a moderately stronger emotional response.

Together with the manual validation results, these aggregates suggest that the extracted fields provide a coherent descriptive profile of brand-level review perceptions. The distribution beneath these averages further sharpens the contrast. As Table 8 shows, Bose reviews disproportionately fall into the strongest advocacy category, while the share of negative recommendations is substantially higher for JBL and Beats than for Bose or Sony, indicating that the gap between the leading and trailing brands extends beyond marginal score differences into the tails of the advocacy distribution.

**Table 8:** Recommendation and emotional connection profile by brand.

Brand	Strongly recommend (%)	Not recommend (%)	Strong emotion (%)	Weak emotion (%)
Sony	47.1	17.8	28.3	32.4
Bose	58.5	15.3	39.9	24.1
JBL	41.0	28.6	28.8	39.8
Beats	45.1	27.5	30.9	38.7

Table 8 also reveals an informative contrast between functional endorsement and emotional resonance. Bose is strong on both dimensions. Sony generates stronger endorsement than affective intensity. Beats retains comparatively greater emotional strength than its quality and value scores would predict. JBL presents the most fragile profile, combining the highest share of negative recommendation with the highest share of weak emotional connection. The perceptual maps extend these aggregate results by showing how the brands occupy different competitive positions. Fig 2 plots mean quality perception against mean value perception. Bose occupies the strongest quality position while maintaining a favorable value score. Sony sits slightly below Bose on quality but records the highest value perception, positioning it near the quality frontier with less price resistance. JBL and Beats cluster in the lower portion of the map; Beats scores somewhat higher than JBL on quality but lower on value, while JBL occupies a more moderate position on both dimensions.

**Fig 2:**
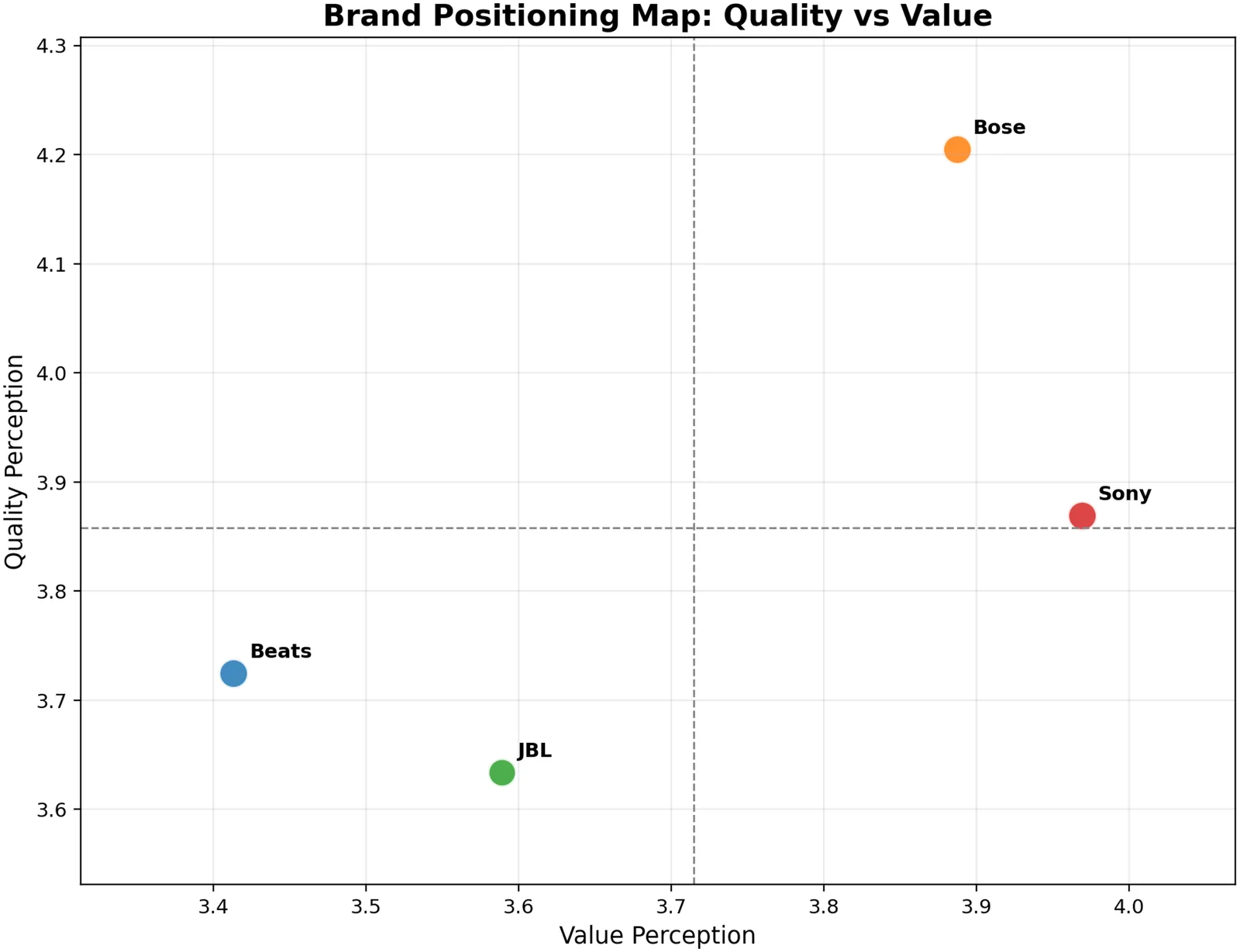
Brand positioning map based on mean value perception and mean quality perception.

The central implication is that premium positioning in this category is not experienced uniformly. Bose commands both quality recognition and resilient value perceptions, whereas Sony benefits from a stronger narrative grounded in functional value. Notably, the map does not yield a simple split between premium and value positioning. Both Bose and Sony occupy the upper region, but for distinct reasons, suggesting that consumers in this category do not regard premium pricing as inherently incompatible with value. Rather, value perception appears to depend on whether the overall performance and use experience are judged to warrant the price. Fig 3 introduces a relational positioning view by plotting recommendation rate against strong emotional connection rate. Bose again occupies the most favorable position, combining the highest advocacy with the deepest emotional bond. Sony remains strong on recommendation but trails Bose on affective intensity, suggesting that consumers endorse the brand more readily than they emotionally identify with it. Beats is notable for generating stronger emotional resonance than Sony and JBL despite weaker functional scores, consistent with the brand’s symbolic appeal and lifestyle reputation. However, this emotional strength is constrained by a high negative recommendation share, indicating that symbolic appeal has limits when price justification is weak. JBL records the lowest recommendation rate and the highest weak-emotion share, confirming a fragile relational profile.

**Fig 3:**
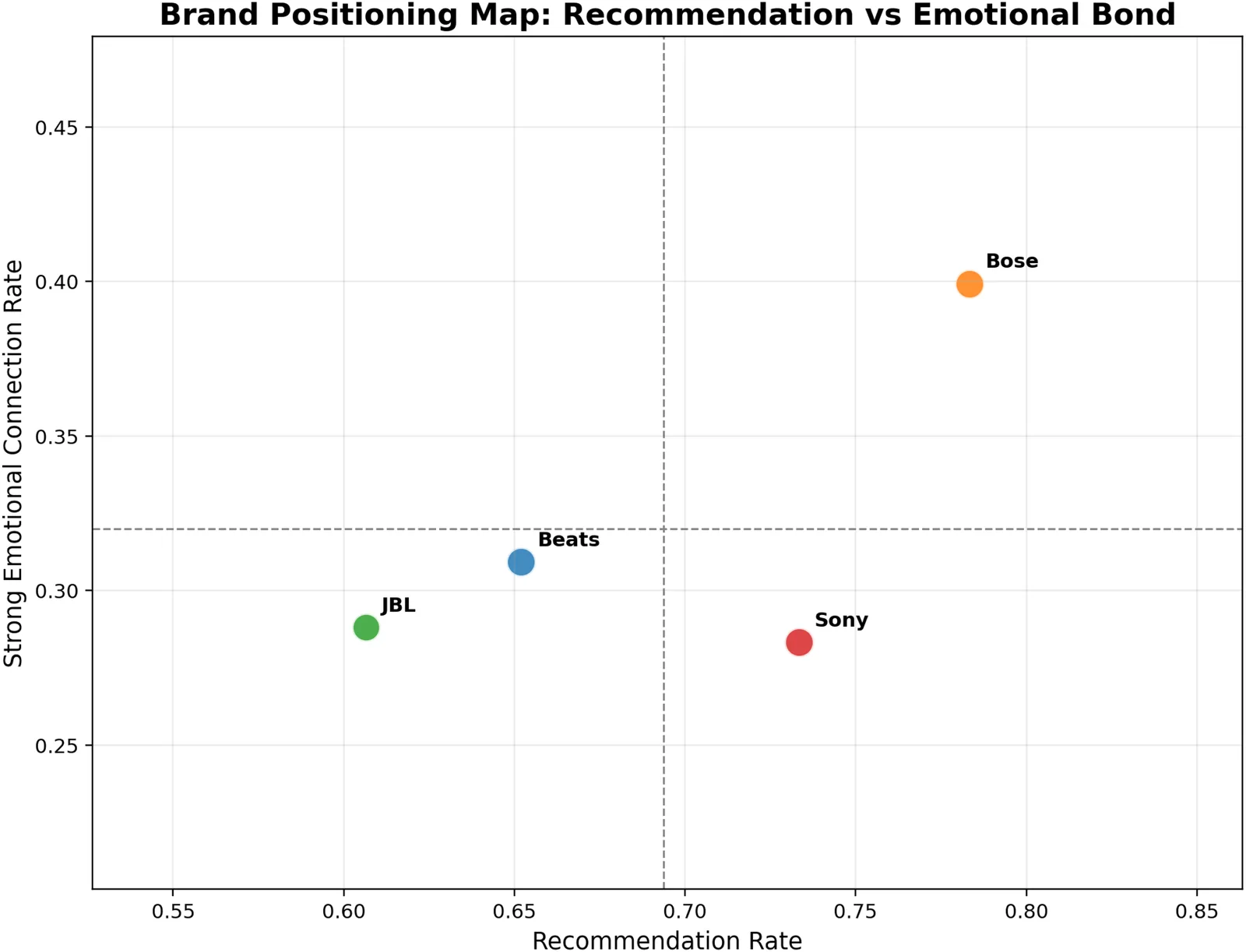
Brand positioning map based on recommendation rate and strong emotional connection rate.

### Association, attribute focus, and dissatisfaction diagnostics

The extracted association labels and attribute categories reveal how consumers interpret each brand. Bose is characterized by a salient association with comfort, while consumer attention is distributed relatively evenly across sound quality, ANC, and comfort. This pattern is consistent with a brand positioned around a holistic listening experience rather than a single technical attribute. Sony is led by associations with reliability, and its high value score suggests a position centered on practical value. Consumer attention toward Sony is concentrated on sound quality, comfort, and value. JBL shows the strongest concentration of attention around sound quality, while its affordable salient association is complicated by recurring negative-review themes related to durability, fit, and consistency. Beats is associated with style, while negative reviews repeatedly question whether its performance justifies the price. Consumer attention toward Beats spans sound quality, value, and design. Table 9 presents these patterns in comparative form.

**Table 9:** Top extracted primary attribute focuses by brand (% of reviews).

Brand	Attribute focus 1	Attribute focus 2	Attribute focus 3
Sony	sound quality (33.4)	comfort (17.7)	value (15.4)
Bose	sound quality (23.9)	ANC (20.6)	comfort (16.7)
JBL	sound quality (42.7)	comfort (15.3)	value (10.8)
Beats	sound quality (30.5)	value (18.2)	design (14.3)

Negative review analysis further differentiates the four brands. As Table 10 summarizes, Bose, despite its overall strength, draws criticism centered on long-term price justification and material degradation. Sony’s negative reviews tend to be more narrowly operational in nature. JBL faces broader consistency challenges, particularly regarding durability and fit. Beats confronts the sharpest tension between price and perceived sound quality, with reviewers repeatedly questioning whether the performance warrants the premium. The relative volume of negative reviews reinforces these patterns: Bose accumulates the fewest dissatisfied experiences in the analytic sample, whereas JBL and Beats exhibit not isolated defects but diffuse dissatisfaction spanning multiple product attributes.

**Table 10:** Recurring dissatisfaction themes from negative reviews.

Brand	Recurring theme	Illustrative issues
Sony	Functional friction	cord too short; no bass at all; insecure 3.5 mm jack; ineffective noise canceling; one side stopped working
Bose	Price justification and material wear	not worth the price; ear cushions deteriorate and flake; fit or connection issues; parts broke after extended use
JBL	Durability, fit, and consistency	one side stopped working; plastic cracked; ear tips fell off; earbuds constantly fall out; awkward heavy fit
Beats	Weak price to performance ratio	poor sound for the price; poor sound quality; not worth the price; cheap sound quality; battery inconvenience

## Discussion

The results, supported by the manual validation exercise, indicate that constrained LLM coding can recover a multidimensional brand perception structure from naturally occurring review text, reinforcing the foundational premise that brand equity operates as a configuration of associations, judgments, and relationship signals rather than as a single evaluative score [1,3]. The validation results are important because they show that the extracted quality, value, recommendation, and attribute-focus variables were not merely unconstrained model outputs, but aligned with independent human judgments at a level suitable for aggregate brand comparison. At the same time, emotional connection should be interpreted as an affective signal rather than as a fully validated measure of brand attachment. This finding extends prior text-based marketing research [4,5] and advances the emerging literature on LLMs in marketing analytics beyond simulated respondents toward comparative analysis grounded in real consumer discourse [11–13]. A central finding is that the four brands occupy distinct and interpretable positions across the extracted dimensions. Bose commands the strongest premium position, combining quality leadership with emotional resonance and high advocacy. Sony achieves comparable advocacy through a different mechanism, grounded in functional value and engineering credibility rather than affective intensity. Beats retains measurable symbolic and emotional appeal despite weaker functional evaluations, while JBL’s profile is marked by concentrated sound quality focus undermined by recurring durability and fit concerns. These patterns suggest that review-based brand perception is best read as a bundle of partially independent signals, consistent with classic brand equity theory [2] but here recovered at scale from marketplace text rather than bespoke survey instruments. For positioning research, this implies that theory-guided LLM extraction can produce continuous perceptual diagnostics from review language, complementing rather than replacing traditional survey-based mapping [18,33].

The methodological value of the workflow lies in the discipline it imposes on LLM output. By enforcing a controlled schema and applying systematic normalization, the extraction process functions closer to a computational coding protocol than to unconstrained generation, addressing the concern that LLM outputs may be too variable for repeated measurement. At the same time, some extracted labels remain broad evaluative summaries rather than precise associations, and free-text dissatisfaction descriptions still require interpretive grouping, underscoring that LLM coding reduces analytical labor but does not eliminate the need for human judgment. A productive design principle emerges: constrain where comparability matters, and allow flexibility where diagnosis matters.

These findings carry differentiated managerial implications. Bose’s priority is defensive, sustaining its comfort and noise cancellation leadership while mitigating the price justification concerns that surface among its small pool of dissatisfied reviewers. Sony should preserve its value-oriented credibility while addressing the operational inconsistencies that erode otherwise positive evaluations. JBL requires consistency improvement more than image amplification, given that durability and fit issues repeatedly undermine its core proposition. Beats needs stronger functional support to counter the persistent perception of overpricing, as design and ecosystem appeal alone prove insufficient to sustain price justification. This differentiated reading illustrates why the framework may offer greater managerial utility than aggregate sentiment dashboards, which tend to flatten distinct problem structures into a single scale. Because the workflow operates on structured extraction from naturally occurring reviews, it could be deployed repeatedly to monitor positioning drift, assess campaign effects, or evaluate whether product changes improve value perception without eroding premium associations.

### Limitations

The findings should be interpreted in light of several limitations. Using a similar number of reviews for each brand facilitated comparison, but it did not ensure that positive, neutral, and negative opinions were represented in their population proportions; alternative sampling or weighting procedures might therefore produce different aggregate estimates. Although filtering removed clear category mismatches, differences among product models, price ranges, and review periods may still influence the observed patterns. The extracted measures also remain dependent on the selected language model, prompt, and coding scheme, despite validation against human judgments. Finally, evidence from four headphone brands may not generalize to other product categories, platforms, markets, or more recent periods.

## Conclusions

This study developed and validated an LLM-driven framework for converting e-commerce reviews into structured brand perception indicators. Applied to 5,895 headphone reviews across Sony, Bose, JBL, and Beats, the framework revealed distinct competitive positions across perceived quality, value, emotional connection, advocacy, and attribute attention. The findings show that reviews can be used not only as indicators of sentiment, but also as evidence of brand associations, evaluative criteria, and sources of dissatisfaction. The study demonstrates how naturally occurring consumer language can support scalable competitive brand diagnosis. The broader implication is that LLMs may be most valuable in marketing research not when they simulate entire markets, but when they help recover structure from the language that markets already generate. Generative AI, in this sense, serves as a bridge between digital trace data and classical brand theory. While the present study focuses on one product category and four brands, it opens two important directions for future research. First, larger-scale validation across product categories would further establish the reliability of LLM-coded brand perception variables across analytical settings [24,33]. Second, future studies could apply the framework across categories and over time to examine how brand perceptions shift around product launches, marketing campaigns, or changes in competitive activity. These directions would move review analysis enabled by LLMs from a category specific diagnostic tool toward a broader approach to AI assisted brand measurement and competitive intelligence.

## Supporting information

S1 Appendix LLM prompt template and reproducibility settings. Full prompt template used for structured extraction, together with the model identifier, decoding parameters, and post-processing procedure.
(PDF)
