# Supplementary material for: Mapping headphone brand positioning from e-commerce reviews with large language models
Source: PLoS One. 2026 Nov 11;21(11):e0361055. doi: 10.1371/journal.pone.0361055 (PMC13754154; doi:10.1371/journal.pone.0361055)
Supplement: Supplementary file 1 [file pone.0361055.s001.pdf]

S1 Appendix. LLM prompt template and reproducibility settings

LLM prompt template

The following prompt structure was used in the extraction script, with the brand name, review text, star rating, and product title inserted dynamically:

```
You are an expert brand perception analyst specializing in
consumer electronics.
Analyze this consumer review of a {brand} headphone product
and extract structured brand perception insights.

Review text: "{review_text}"
Star rating: {star_rating}/5
Product: {product_title}

Return a JSON object with exactly these fields:
{
  "brand_image": "<one word from the controlled vocabulary>",
  "quality_perception": <integer from 1 to 5>,
  "value_perception": <integer from 1 to 5>,
  "emotional_connection": "strong/moderate/weak",
  "recommend_intent":
    "strongly_recommend/recommend/neutral/not_recommend",
  "primary_attribute_focus":
    "sound_quality/comfort/design/value/ANC/durability/
     build_quality/battery/connectivity",
  "dissatisfaction_source": "<specific issue in a few words, or 'none'>"
}
```

LLM configuration and reproducibility settings

To ensure reproducibility, we configured the Large Language Model using specific settings. We selected gpt-5.4 as the model identifier. The input fields consisted of the brand name, review text, star rating, and product title, processed via a fixed zero-shot structured extraction prompt. No separate system-message instruction was used; instead, the role instruction was included directly in the prompt template. The output format was restricted to a JSON object with predefined fields and controlled vocabularies. For generation parameters, the temperature was set to 0, top-p was left at its default, and the maximum output tokens were limited to 300. Regarding post-processing, outputs were parsed as JSON and checked against the predefined schema. Out-of-schema values were normalized using deterministic rules, and non-parseable outputs after retry were handled consistently across all brands.
